# Progressive Adolescent Idiopathic Cervical Kyphosis Secondary to Constant Postural Neck Flexion Reading Habit with a 10‐year Follow‐up: Case Report and Literature Review

**DOI:** 10.1111/os.13356

**Published:** 2022-06-10

**Authors:** Lianlei Wang, Yakubu Ibrahim, Yonghao Tian, Suomao Yuan, Xinyu Liu

**Affiliations:** ^1^ Department of Orthopedics, Qilu Hospital, Cheeloo College of Medicine Shandong University Jinan Shandong P. R. China; ^2^ Cheeloo College of Medicine Shandong University Jinan Shandong P. R. China

**Keywords:** Adolescent idiopathic kyphosis, Case report, Neck flexion reading habits, Progressive cervical kyphosis

## Abstract

**Background:**

Although it has been established that adolescent idiopathic cervical kyphosis (AICK) has no known cause, there are associated risk factors. However, the underlying causes remain puzzling. This case report presents severe AICK linked to chronic neck flexion postural habit, treated with combined anterior and posterior correction surgery and review of the literature.

**Case presentation:**

A 16‐year‐old male with no history of trauma, surgery, or family history of spinal deformity complained of intolerable neck pain and rigidity. He developed an incessant reading of comic books at a very young age, and he preferred placing the book on the floor with his head flexed between his thighs. Acupuncture and massage therapy failed to relief symptoms. He had no neurological symptoms on examination and X‐ray showed Cobb angle of 70.5°. MRI and CT scans showed no spinal cord compression or osteophyte formation. A combined anterior and posterior correction surgery was performed after a week of skull traction. The deformity was corrected, neck pain disappeared, and neck rotatory function maintained after posterior implant removal. The maximum follow‐up was 10 years.

**Conclusions:**

The potential underlying risk factor observed in this case is unusual. Chronic neck flexion postural habit is a potential risk factor of severe AICK in some individuals.

## Graphical abstract

The current case reports an unusual predisposing factor of adolescent idiopathic cervical kyphosis (AICK) of a 16‐year‐old with no history of trauma, surgery, or family history of spinal deformity developed a severe cervical kyphosis. In some individuals, chronic neck flexion postural habit without any comorbidities may be a potential underlying cause of AICK. A detailed history taking is crucial to rule out any likely underlying cause.

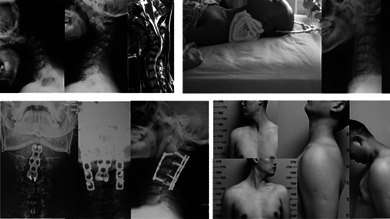

## Introduction

Cervical spine kyphotic deformity occurring in the adolescent without an identifiable cause is often referred to as AICK. A severe cervical kyphotic deformity (CKD) often results in a catastrophic functional disability such as problems with forwarding gaze and dysphagia. Consequently, the patient's health‐related quality of life (HRQoL) can be jeopardized in a myriad of ways. The causes of CKD can be congenital, iatrogenic, or idiopathic. Here, we present an AICK case linked to chronic neck flexion postural habit in a 16‐year‐old. Though previous studies have associated neck flexion habits and neck extensor muscles with the development of CKD,^1^, ^2^ this is the first report indicating neck flexion postural reading habit as a potential underlying cause of severe AICK without any trace of preexisting conditions. The current case report was drafted following the CARE guidelines.

## Case Presentation

A 16‐year‐old male Chinese presented with severe neck pain, rigidity, and obvious cervical deformity. His height and weight were 175 cm and 58 kg (BMI 18.9 kg/m^2^) and the visual analogue scale (VAS) for neck pain was 9.0 on admission. The underlying cause was linked to his unusual postural reading habit. As most kids do in this part of the world, he developed a strong passion for reading. He started reading comic books as soon as he could read and understand language. Although the specific number of hours spent reading could not be quantified, his mother estimated 6 h of reading time daily. He seemed to be comfortable reading in his unusual postural habit. He was left alone to go on with his reading posture because both his parents and teachers alike were afraid of discouraging him from reading.

He preferred sitting and placing the book on the floor with his head and neck in a flexion position between his thighs instead of sitting in a chair. All efforts by his parents to modify his postural reading behavior were futile. They were oblivious to the gradual changes of his cervical spine until the deformity became obvious to ignore. The deformity affected his forward gaze, his neck could no longer return to a neutral position at will affecting his ability to walk safely, and the unbearable neck pain ensued almost rendered him incapacitated. His neck pain exacerbated in flexion posture. The pain became constant and the acupuncture, massage treatment he received before reporting to the hospital only provided episodic pain relief. He had no history of trauma, surgery, or a family history of spinal conditions.

### Pre‐Operative Course

Severe cervical kyphotic deformity, thick, and tight neck muscles were noticed on examination. He was neurologically intact and his deep tendon reflexes were normal. Assessments for possible muscle hypertonia/hypotonia, laxity of joints, mental disorder were negative.

**Fig. 1:**
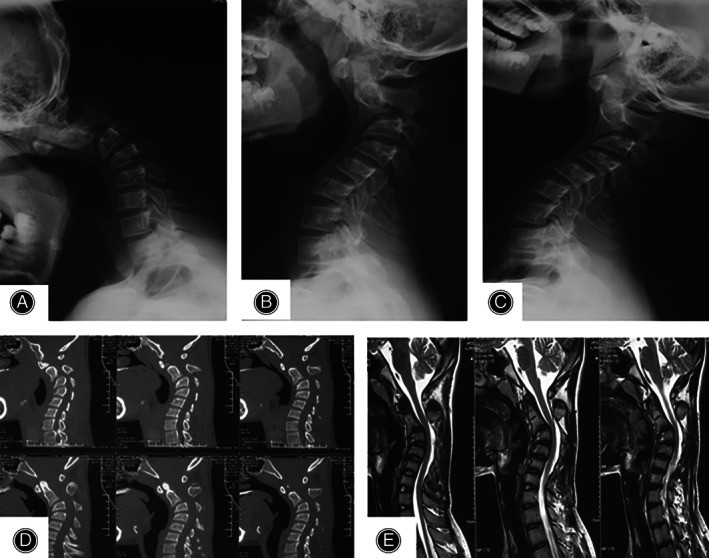
Preoperative radiology: lateral flexion(A), neutral(B), and extension(C) showing a cervical kyphotic deformity. Sagittal CT scan(D) showing the kyphotic bony anatomy with no sign of osteophyte formation. Sagittal MRI(F) showing no obvious cord compressing.

A conventional X‐ray indicated cervical spine flexion deformity with Cobb angle of 70.5°, 87.6°, 51.8° (Fig. 1A–C) on neutral, hyperextension, and hyperflexion respectively.

CT scan revealed the bony anatomy of the cervical spine with no obvious osteophyte formation (Fig. 1D) and MRI findings were normal without any significant spinal cord compression (Fig. 1E).

An angiography was obtained to ascertain any potential vertebral artery and other vascular anomalies to prevent vascular trauma during surgery. Vascular anatomical anomalies were ruled out after evaluation.

**Fig. 2:**
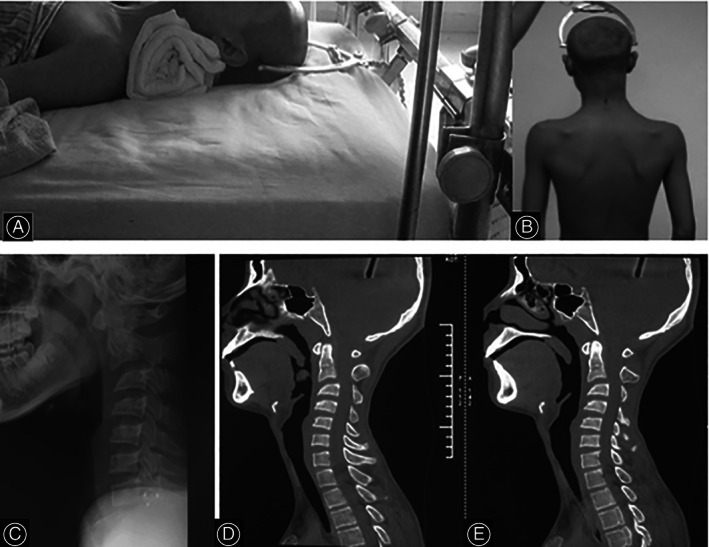
Preoperative skull traction application (A, B); lateral x‐ray (C) and sagittal CT scan (D and E) showing a significant reduction of the cervical deformity after a week intervention.

Before surgery, skull traction (5 kg) was applied for 7 days and a significant reduction in the kyphosis was obtained Cobb angle of 33.9° (Fig. 2). However, the patient developed mild pressure sores and could not tolerate further traction even if it was necessary. His preoperative VAS score was 7.

### Operative Course

A week after cervical traction, an anterior fusion, and posterior screw fixation were performed to correct the deformity.

An electro‐physiological spinal cord monitoring was installed intraoperatively to observe motor‐evoked potentials. After a single general anesthetic, the anterior fusion was performed first. The C2‐5 intervertebral disc was resected and removed. A subtotal resection of C3 vertebral body was performed, cortical bone harvested from the iliac crest was grafted into the C2‐C4 and C4‐5 intervertebral spaces, and correct sized titanium with screws was installed. A posterior C2 pedicle screw and C3‐C5 lateral mass instrumentation were performed after the anterior fusion.

**Fig. 3:**
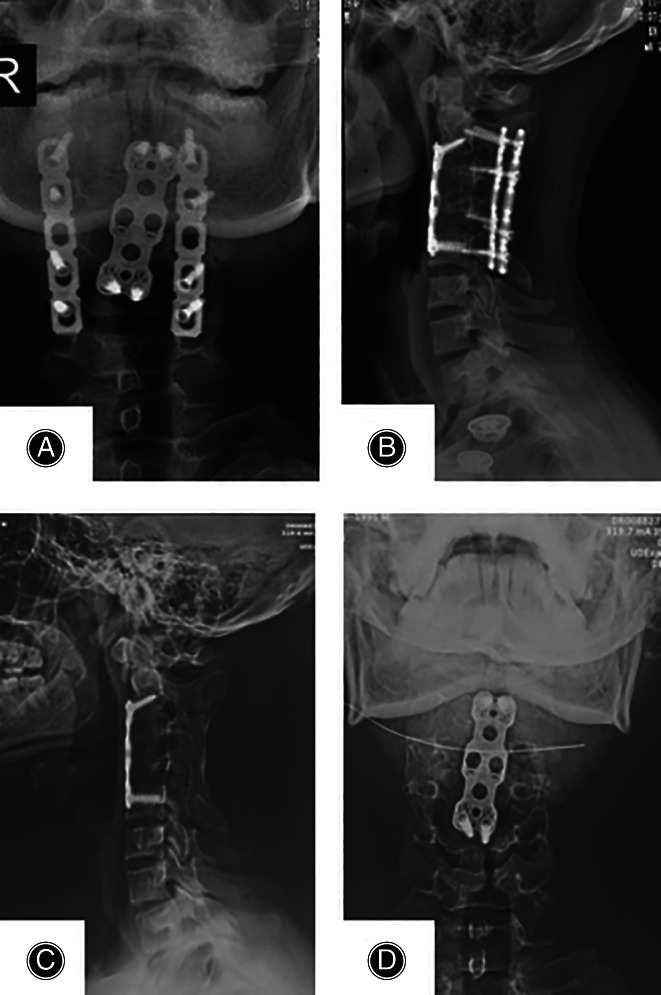
Conventional radiology: Postoperative AP and lateral view at 2.5‐year before posterior implant removal (A and B) showing cervical alignment and bone union; AP and lateral radiographs at the last follow‐up indicating an optimal cervical spine correction and bone union (C and D).

The general anesthesia was successfully reversed, the patient was extubated on the operation table, and did not require intensive care. Postoperative radiology showed a Cobb angle of 17.6 (Fig. 3B). His pain improved significantly with a VAS score of 1 at POD 2.

A Philadelphia collar was put in place for neck support, and he was able to sit up straight in bed without neck pain. He was discharged without complications.

### Post‐Operative Follow‐up Course

His VAS score at 3 months follow‐up was 0, the Cobb angle on X‐ray at 30 months follow‐up was 18.2° (Fig. 3C), and the posterior internal fixation was removed after a satisfactory bone fusion was achieved with improved cervical sagittal alignment.

At eight and a half years follow‐up, the patient was in perfect health with no discomfort or disability with a Cobb angle of 18.6° and complete bone fusion was attained.

**Fig. 4:**
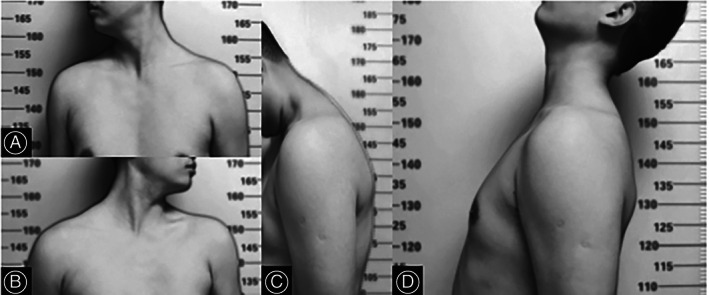
Images at the last follow‐up 10 years after surgery: (A–D) showing right neck rotation, flexion, left rotation, and neck extension respectively with a great painless ROM.

The last follow‐up was at 10 years after the surgical deformity correction and the patient was doing well in all aspects of life and he still enjoyed reading with modified reading habits. The VAS score at the last follow‐up was 0. He had a great cervical range of motion with left and right neck rotatory function of 90°, 55° on flexion, and 45° on an extension(Fig. 4). Further radiological assessments were then discouraged to reduce potential radiation exposure and avoid unnecessary financial burden.

## Discussion

Cervical kyphosis can be progressive once triggered, resulting in a gruesome neurological symptoms.^3^ Treatment modalities for cervical kyphosis are often reported in the literature. Severe and progressive AICK reports are, however, rare (Table 1). A mild to moderate AICK can be resolved without any rigorous intervention. However, unresolved progressive deformity, often observed in spondylitis arthropathies can result in a chin‐on‐chest phenomenon disrupting swallowing, breathing, and horizontal gaze.^4^, ^5^

**TABLE 1:** Summary of adolescent idiopathic cervical kyphosis diagnosis and treatment

Study	Age(y)/sex	Cause of deformity	ULC	C/C	Pre‐op angle (°)	Post‐op angle (°)	Max. FU(y)	Pre‐op traction	Post‐op complications	Treatment
Iwasaki *et al*., 2002^20^	11, M	Idiopathic	NM	Neck pain	70°	NM	11	Yes	None	Subtotal corpectomy
Iwasaki *et al*., 2002^20^	11, F	Idiopathic	NM	Neck pain, restricted ROM	75°	26°	6	Yes	None	Subtotal corpectomy
Saito *et al*., 2013^1^	18, F	Schizophrenia	Postural habit	Neck pain, restricted ROM	109°	23°	2	Yes	Right shoulder abduction weakness	Combined anterior/posterior procedure.
Yamamuro *et al*., 2019^21^	15, F	Idiopathic	NM	Neck pain	17°	0°	1	Yes	None	Combined ant/post. Surgery
Present case	16, M	Idiopathic	Chronic neck flexion habit	Neck pain	70°	17.6°	10	Yes	None	Combined ant/post. Procedure

C/C, chief complain; Max. FU, maximum follow‐up; NM, not mentioned; post‐op, post‐operation; Pre‐op, pre‐operation; ROM, range of motion; ULC = underlying cause.

There are many factors and causes reported to be associated with the development of cervical spine kyphosis. They include but not limited to trauma, neuromuscular disease, psychiatric condition, ankylosing spondylolitis and post‐laminectomies.^6^, ^7^, ^8^, ^9^

The most common iatrogenic cause of CK is secondary to cervical surgery,^10^ which may be linked to post‐surgery pseudoarthrosis or distortions in the natural cervical lordosis. It has been observed that young adults and the pediatric population are susceptible to postlaminectomy cervical kyphosis (PLCK).^11^, ^12^ One reason for this trend is associated with the less developed bony and ligamentous structures of the cervical spine. In contrast, PLCK is uncommon in adults partly due to the well‐developed bony and ligamentous structures of the spine.

The phenomenon may explain why a constant neck flexion habit predisposes the young to suffer AICK as observed in the current report. The accumulative effect of certain mundane habits leading to a grim cervical spine deformity, such as AICK encountered in this case, is often missed, or ignored entirely. Therefore, a detailed history of the patient presented with AICK is crucial to rule out any potential underlying cause.

The exact causes of AICK remain unknown, but the predisposing factors and potential underlying causes are undeniable. As the search continues to pin down the direct or indirect causes of cervical spine deformity in the adolescent, Saito *et al*.^1^ reported AICK in a schizophrenic patient which they associated the cause of the deformity to her postural habit. The interplay between the schizophrenia and the postural disorder leading to AICK in their report cannot be undermined. In this report, we diagnosed a severe AICK triggered and progressed by chronic neck flexion reading habit. Unlike the previous reports, there was no comorbidity involved.

A chronic neck flexion behavior weakens the neck extensor muscles over time resulting in cervical spine kyphosis.^2^ This is consistent with the present report as the patient's history revealed his constant neck flexion reading habit as the potential predisposing factor to his cervical spine deformity.

Once the process of kyphosis development is initiated, there is propensity for progressing due to axial load shift and the pressure exerted on the cervical spine.^13^ A kyphotic spine interrupts the natural alignment of the head in relation to the spine transmitting abnormal forces, consequently, deformity progression.^6^

A study reported that the trapezius muscle and other neck extensors such as the splenius capitis and semispinalis capitis play a crucial role in strengthening and supporting the neck. As a result, a constant neck flexion gradually weakens these supporting muscles of the neck due to overstretching.^14^

In line with the role of the neck muscles in strengthening the cervical spine, a patient with an unknown cause of a 43.3° cervical kyphosis who could not comply with cervical traction treatment, had a complete deformity resolution in 8 months with neck muscles strengthening exercise alone.^2^

Though these are isolated cases, it is suggested that unusual postural habit modifications and neck extensors strengthening exercises may curb AICK development when identified early. Furthermore, a randomized control trial studied patients cervical lordotic deformity and found out that neck extensor exercises significantly improved the lordosis and pain.^15^

Once the neck extensor muscles are weakened either by physical stress or certain medications, cervical spine kyphosis can occur. On drugs effects on the neck extensors, Hogan *et al*.^16^ reported a progressive cervical kyphotic case secondary to botulinum injection into the neck muscles towards neck pain and spasms management. A few days after the botulinum injection, neck weakness ensued, and the patient was unable to extend her neck and developed cervical kyphosis.

These findings bring to light the neck supporting muscles and habitual neck flexion role in the development of CKD, making a valuable contribution to our understanding of this devastating condition. Still, what makes some individuals prone, solely on postural neck flexion habits, to AICK without pharmacological, psychological, or genetic influences is yet to be discovered. However, identifying abnormal neck flexion habits, that overstretch the neck extensors, early while initiating mild to moderate neck exercises in patients with mild AICK may correct the deformity without rigorous treatment or surgery.

Even though the cause of AICK remains unknown, the current report and a few studies in the literature^1^, ^2^, ^17^ suggest that constant neck flexion can orchestrate the development of cervical kyphotic deformity.

The predominant initial treatment options, that aim at alleviating symptoms have been conservative maneuvers such as brace neck immobilization, physiotherapy, chiropractic maneuvers, and medications.^18^ However, conservative treatment is often reserved for patients with no neurological lesions, myelopathy, and with a flexible deformity.

Surgical correction is recommended for patients with debilitating progressive symptoms after conservative management has failed.

A significant deformity reduction was achieved with a skull traction in a week (Fig. 1D–F).

Although the radiography evaluation did not review any significant sign of cord compressing or osteophyte formation (Fig. 1D and E), the present patient was a candidate for surgical treatment due to the progressive nature of the kyphosis and the associated severe neck pain.

Several surgical correction methods have been described after Mason *et al*.^19^ first performed anterior cervical osteotomy approach to correct cervical spine deformity. Still, there is no ideal surgical approach for the treatment of CKD.

As applied for the present case, ACDF and subtotal resection was effective approach for the AICK treatment; the patient had a tremendous recovery without any complication, achieved a complete bone union, and the neck rotation function was impeccable after the posterior implant removal (Figs 3 and 4).

The last follow‐up was 10 years after the procedure and the patient was doing well with no surgery‐associated sequalae.

The risk factors and surgical management approaches of severe AICK have been established. As mentioned above, a handful of studies have reported the potential association between neck extensor muscles weakness, postural habits, and cervical spine deformity occurrence. To our knowledge, however, this is the first report that describes neck flexion postural reading habit as the sole potential underlying cause of AICK.

## Conclusion

A chronic neck flexion postural habit in normal patient without comorbidities, may be a potential underlying cause of AICK in some individuals. The underlying cause presented in this case is distinct and highlights the significance of detailed history taking to rule out or in any underlying cause of this devastating condition. Given the significant constraints of a single case report, any conclusion taken from this instance should be regarded as provisional.

## Conflict of interest

All authors declare that they have no competing interests.

## Funding information

This work was supported in part by the National Natural Science Foundation of China (81874022 and 82172483 to Xinyu Liu; 82102522 to Lianlei Wang) and Natural Science Foundation of Shandong province (ZR202102210113 to Lianlei Wang).

## Data Availability

The datasets used for the current case report are available from the corresponding author on reasonable request.
